# Abscopal effect: from a rare phenomenon to a new frontier in cancer therapy

**DOI:** 10.1186/s40364-024-00628-3

**Published:** 2024-09-04

**Authors:** Xueying Wang, Haoyu Zhang, XinZhang, Yong Liu

**Affiliations:** 1 Department of Otolaryngology Head and Neck Surgery, Xiangya Hospital, Central South University, 87 Xiangya Road, Changsha, 410008 Hunan People’s Republic of China; 2 Otolaryngology Major Disease Research Key Laboratory of Hunan Province, 87 Xiangya Road, Changsha, 410008 Hunan People’s Republic of China; 3 Clinical Research Center for Laryngopharyngeal and Voice Disorders in Hunan Province, 87 Xiangya Road, Changsha, 410008 Hunan People’s Republic of China; 4 National Clinical Research Center for Geriatric Disorders (Xiangya Hospital), Changsha, 410008 Hunan China

**Keywords:** Abscopal effect, Radiotherapy and immune therapy, Radiotherapy, CD8 + T, Immunotherapy

## Abstract

Radiotherapy (RT) controls local lesions, meantime it has the capability to induce systemic response to inhibit distant, metastatic, non-radiated tumors, which is referred to as the “abscopal effect”. It is widely recognized that radiotherapy can stimulate systemic immune response. This provides a compelling theoretical basis for the combination of immune therapy combined with radiotherapy(iRT). Indeed, this phenomenon has also been observed in clinical treatment, bringing significant clinical benefits to patients, and a series of basic studies are underway to amplify this effect. However, the molecular mechanisms of immune response induced by RT, determination of the optimal treatment regimen for iRT, and how to amplify the abscopal effect. In order to amplify and utilize this effect in clinical management, these key issues require to be well addressed; In this review, we comprehensively summarize the growing consensus and emphasize the emerging limitations of enhancing the abscopal effect with radiotherapy or immunotherapy. Finally, we discuss the prospects and barriers to the current clinical translational applications.

## Introduction

Radiotherapy (RT) is a crucial treatment option for tumors, with approximately 50% of cancer patients receiving this therapy [1, 2]. RT induces an irreversible DNA damage and has a direct cytotoxic effect on cancer cells. Recently, RT’s immune modulatory effects on the tumor microenvironment (TME) and surrounding normal tissue have garnered significant attention [3–5]. The immune modulatory effect of RT paves a novel avenue for the anti-tumor response. Localized RT promotes the immune-mediated control of distant metastases, which is defined as the abscopal effect [6]. Unfortunately, this effect is not frequently observed in clinical practice, due to the facts that immunosuppressive TME or insufficient activation of the anti-tumor response [4]. Immunotherapy has achieved substantial advancements in the treatment of advanced or late cancer patients, especially with the utilization of anti-CTLA-4 and anti-PD1/anti-PDL1 monoclonal antibodies. RT can also induce immune system activation, suggesting that RT can also elicit an immune-mediated anti-tumor response [7]. Therefore, the immune-modulating effects of RT provide a potential for synergistic effects with combination immunotherapy, which is referred to as immune therapy combined with radiotherapy(iRT).

Tumor immunotherapy has become one of the most successful treatment modalities, particularly in the therapy of solid cancers using immune checkpoint inhibitors (ICIs) [8].

From a mechanistic perspective, ICIs, one of the most important cancer immunotherapeutic strategies in clinical practice, activate anti-tumor immune responses and enhance immune-mediated malignant cell elimination by blocking these immune checkpoints [9, 10]. Initially, PD-1 is expressed on the surface of T-cells and is intricately linked to programmed cell death [11]. PD-1 binds with its ligand PD-L1, then exerts negative effects on anti-tumor immunity via the inhibition of TCR-mediated cytokine secretion and lymphocyte proliferation [12–14]. It is puzzling that tumor cells also overexpress PD-L1 to evade immune surveillance [15, 16]. In fact, multiple monoclonal antibodies have been conferred benefits upon a subset of patients [17, 18]. Meanwhile, CTLA-4 is a co-inhibitory molecule expressed on T cells, which inhibits the proliferation and activation of T cell [19, 20]. Through clinical trials and efficacy evaluations, ipilimumab, a CTLA-4 monoclonal antibody, has become the first ICIs approved for cancer treatment, which stimulates effective immune responses while suppressing tumor progression [18, 21]. However, only a small fraction of patients exhibit sustained responses to ICIs [22]. Therefore, expanding the beneficial scope of immunotherapy and accurately identifying patients with higher sensitivity are urgent issues that need to be addressed. Increasingly, studies have shown that iRT can potentially overcome the aforementioned issues. Specifically, radiation-induced tumor cell death releases neoantigens that enhance the immune system’s recognition of tumor cells. At the same time, radiotherapy can change the tumor microenvironment, reduce the activity of immunosuppressive cells, and improve the penetration and activity of immune cells. Finally, radiotherapy combined with immunotherapy contributes to the formation of long-term immune memory, thereby improving the anti-relapse potential. In the following review, we will make a more specific explanation of the above content [23, 24].

iRT has yet to achieve groundbreaking progress, however, numerous issues remain to be resolved in future. Primarily, the abscopal effect was predominantly recognized, yet the impact of iRT on the TME and the potential mechanism by which it induces abscopal effects on tumors remains elusive [25, 26]. On the other hand, the likelihood of abscopal effects occurring in clinical practice is relatively low, and we should consider how to amplify and employ this effect in clinical settings to control metastatic lesions [27]. Radiation therapy and immunotherapy indeed confer substantial benefits to cancer patients. However, considering that some patients do not benefit or the effects are not pronounced, we have summarized and expected the potential clinical applications and optimal combination strategies for iRT, including the mechanisms of iRT in combination therapy, radiation dosage, and treatment sequencing.

## The development of radiotherapy and immunotherapy

Radiotherapy (RT) has evolved significantly since its discovery, progressing through various technological advancements that have improved its efficacy and safety. Similarly, immunotherapy has a long history, with recent breakthroughs in immune checkpoint inhibitors (ICIs) offering substantial benefits for cancer treatment. The combination of RT and immunotherapy (iRT) leverages the strengths of both approaches to enhance anti-tumor responses [28]. In 1896, a breast cancer patient was the first to receive X-ray RT [29]. Although X-rays have been used for cancer treatment for decades, the molecular mechanisms and biological effects are still not fully understood. Regaud and Coutard have reported that step therapy can offer a cumulative radiation dose while also providing significant clinical benefits [30]. The discovery of the X-ray tube greatly propelled the development of X-rays, as it could promote the Compton effect and provide X-rays with higher energy (180–200 kV) [31]. From 1930 to 1950, this was a period of vigorous development in physics and machines, which we define as the positive voltage era. Lawrence and Livingston invented the cyclotron in 1930^32^. In 1952, the Brookhaven National Laboratory welcomed its first synchrotron accelerator [32]. From 1950 to 1985, it developed into the megavoltage era. The linear electron accelerator and cobalt remote therapy are considered a revolution in cancer treatment, as they generate high-energy radiation to treat deep tissue tumors [33]. By the 1970s, the linear accelerator had been widely used in clinical settings. With the advancements in computer and imaging technologies, radiation therapy has entered its fourth era of development. Computerized tomography and various imaging modalities contribute to precise treatment planning, allowing for accurate identification of tumor localization and surrounding anatomical structures.

**Fig. 1:**
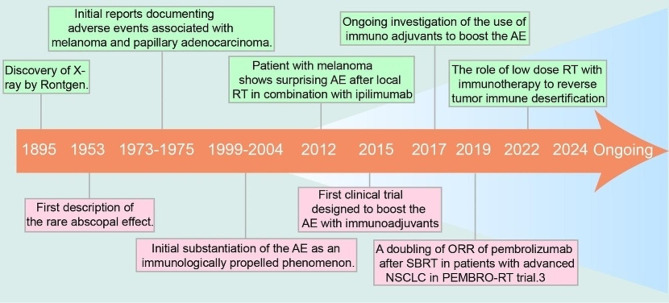
Timeline of significant historical events concerning the abscopal effect

Tumor immunotherapy has a history of more than 100 years [34]. In 1796, Edward Jenner used a vaccine to treat smallpox patients, which laid the immunological foundation for immunotherapy. In 1863, Virchow found the connection between tumors and intratumoral inflammation [35]. In 1891, William Coley, the father of cancer immunology, used Coley toxins to cure the first case of cancer [36, 37]. In 1959, the inaugural study on tumor vaccination, spearheaded by Ruth and John Graham, attained triumph and sparked fervent fascination in this realm. From 1987 to 1995, the preliminary confirmation of CTLA-4 and its immune checkpoint function took place. It was not until 2010 that ipilimumab, as the pioneering checkpoint inhibitor, obtained FDA approval for the clinical management of stage IV melanoma after extensive research. Thereafter, clinician and researchers formed the conception that using the host immune system to fight against cancer, initiated a brand-new era of cancer immunotherapy. In 2013, the journal Science proclaimed immunotherapy as the “breakthrough of the year” for treating tumors. Many reviews have summarized the therapeutic patterns based on iRT amplifying the distal effects. Here, we have compiled them into a temporal evolution diagram (Fig. 1).

Above, we have comprehensively reviewed the development histories of radiotherapy and immunotherapy respectively, which provides a theoretical basis for their combined application to produce abscopal effects.

## The development of abscopal effect and combination of radiotherapy and immunotherapy

Originally introduced by Mole in 1953, the ‘abscopal’ term describes a radiation response mediated by tumour cells that reside distant from the irradiated region [25]. Several cancers have been reported to have the abscopal effect, including melanomas, renal cell carcinomas, breast cancers, hepatocellular carcinomas, and other metastatic solid tumours [38, 39]. Between 1969 and 2014, 46 cases of the abscopal effect have been reported as the result of radiotherapy alone, according to a recent review [27]. From 1973 to 1975, initial case reports of the abscopal effect were reported for melanoma and papillary adenocarcinoma [40, 41]. A study in 1979 showed that ionizing radiation kills cells in part through immune mechanisms, with the host immune system’s integrity determining a tumour’s radiosensitivity [42]. A syngeneic mouse model of fibrosarcoma was used to determine the radiation dose required to control tumour growth. A study found that mice with T cell competence required a lower radiation dose to control tumour growth than mice without T cells. As well, T cell-deficient mice had a greater likelihood of developing metastases, demonstrating a correlation between immune status, local response to radiotherapy, and metastasis.

Numerous studies have since demonstrated a direct link between the abscopal effect and immune system mechanisms [43–45]. For example, an experimental set-up was established for studying the abscopal effects of brachytherapy by sparing a distant site resembling a metastasis when irradiating mouse tumors. The results showed that transplanted MC38-derived tumors only displayed abscopal effects when brachytherapy was combined with immunostimulatory antibodies against PD1 and/or CD137 [46]. Furthermore, in a clinical trial, there was an abscopal response when radiotherapy was combined with granulocyte-macrophage colony-stimulating factor (GM-CSF) for the treatment of multiple types of confirmed solid metastatic cancer [47]. Similarly, radiotherapy combined with antibodies against CTLA4 and PDL1 increased abscopal response rates in a study on melanoma [42]. Numerous studies are currently investigating how radiotherapy and immunotherapy can be combined to improve treatment outcomes for different indications.

## Rationale for the combination of radiotherapy and immunotherapy

The mechanisms by which RT kills tumors, mainly include the direct damage and induction of anti-tumor immune responses [1, 48–50]. Theoretically, RT reshapes the TME and then affects the efficacy of RT, which also builds the foundation for the combination therapy of RT and ICIs. ICIs specifically block the immune checkpoints to enhance the anti-tumor immune responses of CD8 + T cells [51, 52]. Except for the direct killing effect of cancer cells, RT also wields the weapon of host immune system to fight against cancer, such as activating various immune cells [53, 54], enhancing the release and presentation of tumor-specific antigen [55, 56], increasing the density of tumor-infiltrating lymphocytes [3, 57] and so on. In addition, RT changes the extracellular components in TME including chemokines, cytokines, metabolites, blood and lymphatic vessels etc. [49, 58]. TME reprogram induced by RT exerts a “cold-to-hot” effect, switching a “cold” tumor with few infiltration of immune cells into a “hot” tumor with abundant intratumoral lymphocytes, providing an excellent condition for a good response of ICIs therapy [5, 59]. However, we have to mention that in certain circumstances, RT also generates immune suppressive effects that are not beneficial for cancer control [60, 61]. In general, RT-induced immune suppression is divided into two aspects: the death of immune cells in peripheral blood and the increase of inhibitory immune cells (Bone marrow-derived suppressor cells and Treg cells in the TME) [6, 62]. Next, we will review in detail the effects of RT on different cells in the tumor microenvironment. We will next review in detail the effects of RT on different cells in the tumor microenvironment, which lay the foundation for the generation of abscopal effect.

### Effects of RT on cancer cells

RT generates DNA damage through high-energy photons and generating reactive oxygen species(ROS) in cancer cells, leading to the death of cancer cells and enhanced immunogenicity [63, 64]. RT triggers stress responses in apoptotic cancer cells, leading to the release of damage-associated molecular patterns (DAMPs). These DAMPs have potent immunomodulatory effects that affect the behavior of immune cells [65, 66]. Furthermore, the immunogenicity of tumor cells subjected to irradiation can be modified by the cellular reaction to DNA damage provoked by RT. The interplay between the DNA damage response and STING signaling within the tumor cells is critical in the formation of an inflammatory microenvironment that is governed by RT [67, 68]. This process generates the abscopal effect after radiotherapy, which involves the regression of untreated tumors at distant sites from the irradiated tumor site.

The immune system can recognize damaged DNA from various sources, which are exposed to the cytoplasm in cancer cells due to RT [69]. It is believed that immune responses can be triggered by mtDNA damage and ROS caused by RT, with mtDNA damage being more sensitive to DDR disorders than nuclear DNA damage [70]. Whereas, the mechanism underlying mtDNA damage induced by RT is still unclear. Regardless of the source, cGAS can recognize all types of damaged DNA and form a 2:2 complex with the DNA [71–73]. Following the recognition of cytoplasmic DNA, cGAS, acting as a catalyst, facilitates the synthesis of cGAMP [74, 75]. Activation of the cGAS-STING pathway by the binding of 2′3′-cGAMP leads to the stimulation of STING and its subsequent translocation to the Golgi apparatus, followed by the activation of TBK1^76, 77^. This results in the phosphorylation of STING and promotes the translocation of IRF3 to the nucleus. Subsequently, TBK1 phosphorylates the transcription factor IRF3, leading to its activation and translocation to the nucleus, where it binds to the promoter region of the IFN-β gene, triggering its transcription and translation into type I interferons. These interferons are vital immunomodulatory factors that exert a pivotal role in the eradication of cancer cells, by acting as the indispensable bridge between innate and adaptive immune responses mediated by dendritic cells (DCs) [78, 79]. Secretion of Type I interferons by cancer cells helps in the maturation of DCs, increases the expression of co-stimulatory molecules, and improves their ability to migrate to lymph nodes [80, 81].

In the context of RT, the cGAS-STING pathway induces the expression of type I interferons through the activation of multiple pathways, including the canonical and non-canonical NF-κB pathways, as well as IRF-3 [82, 83]. In addition to the generation of ROS, radiation-induced DNA damage can activate the NF-κB pathway through the IKK-dependent canonical pathway [84]. In addition, the expression of IFN-β is heavily reliant on the enhancing effect of the enhanceosome [85, 86]. The canonical NF-κB pathway can collaborate with IRF-3 and other enhancer components to maximize the expression of the IFN-β gene. Furthermore, the enhanceosome is crucial for the expression of IFN-β, as exemplified by the synergistic action of IRF-3 and other enhancers working in concert with NF-κB to facilitate IFN-β expression [87, 88]. Overall, the cGAS-STING pathway, in coordination with multiple pathways including the canonical and non-canonical NF-κB pathways and IRF-3, plays a crucial role in inducing the expression of type I interferons in response to RT. Several studies have indicated that NF-κB pathways play a vital role in the induction of type I IFN expression in response to RT. Abe et al. found that silencing the expression of canonical NF-κB in mice embryonic fibroblasts led to a 50% inhibition of IFN-β production [76]. Moreover, in response to RT-exposed cancer cells, the non-canonical NF-κB pathway can be activated in DCs, leading to the suppression of IFN release [89]. These findings suggest that understanding the interplay between these pathways is critical in the development of immunomodulatory strategies for cancer treatment.

### Effects of RT on stromal cells

Stromal cells are one of the most abundant cell types in the TME, mainly include fibroblasts, pericytes, endothelial cells, immune cells, inflammatory cells and so on and play a crucial role in tumor growth, metastasis, and treatment response [90–92].

Fibroblasts are one of the most common cell types in the TME, and they maintain the structure and function of the TME by synthesizing and secreting matrix components such as collagen and fibronectin [93–95]. The effect of radiotherapy on fibroblasts can be divided into two aspects: direct and indirect. Directly, radiotherapy can induce DNA damage and apoptosis of fibroblasts, leading to the death of fibroblasts and the reduction of extracellular matrix secretion [96–98]. In addition, radiotherapy can activate the TGF-β signaling pathway in fibroblasts, promoting the secretion of extracellular matrix and increasing the stiffness of the TME, which facilitates cancer cell invasion and metastasis [99, 100]. Indirectly, radiotherapy can cause damage to nearby endothelial cells, leading to the secretion of various cytokines and chemokines, which can recruit fibroblasts to the site of injury and promote their activation [101, 102]. Activated fibroblasts, also known as cancer-associated fibroblasts (CAFs), can secrete various factors, including growth factors, cytokines, and chemokines, which promote tumor growth, invasion, and metastasis [103–105].

Endothelial cells are a type of cell that lines the interior surface of blood vessels and plays a crucial role in angiogenesis [106, 107]. Radiotherapy can cause damage to endothelial cells, leading to the secretion of cytokines and chemokines, promoting the recruitment of immune cells and fibroblasts, and inducing angiogenesis [101, 108]. In addition, radiation-induced endothelial cell damage can also lead to vascular leakage, which facilitates the infiltration of immune cells and chemotherapeutic agents into the tumor tissue [109]. These effects can contribute to radiation-induced vascular damage, which can have various clinical implications. For example, radiation-induced vascular damage in normal tissues can lead to side effects such as skin changes, mucositis, and fibrosis [110–113]. In addition, radiotherapy can impact tumor angiogenesis and microenvironment, which can affect tumor growth and response to treatment [108, 114]. Therefore, understanding the effects of radiotherapy on endothelial cells is important for developing strategies to minimize radiation-induced vascular toxicity and improve treatment outcomes.

### Regulation of various immune cells by radiotherapy

#### T cell

T cells are critical components in the adaptive immune system, which can recognize and eliminate cancer cells. The effects of radiotherapy on T cells have been extensively investigated, and the results are complex and often depend on the dose, fractionation, and timing of radiation [54, 56, 115]. In the early stage of radiotherapy, T cell activity may be suppressed due to immune system damage [116, 117]. Some studies have shown that after radiotherapy, T cells exhibit subclinical manifestations, including an increase in regulatory T cells (Tregs) and a decrease in cytokine production [118–120]. These changes may have adverse effects on the efficacy of immunotherapy [121–123].

**Fig. 2:**
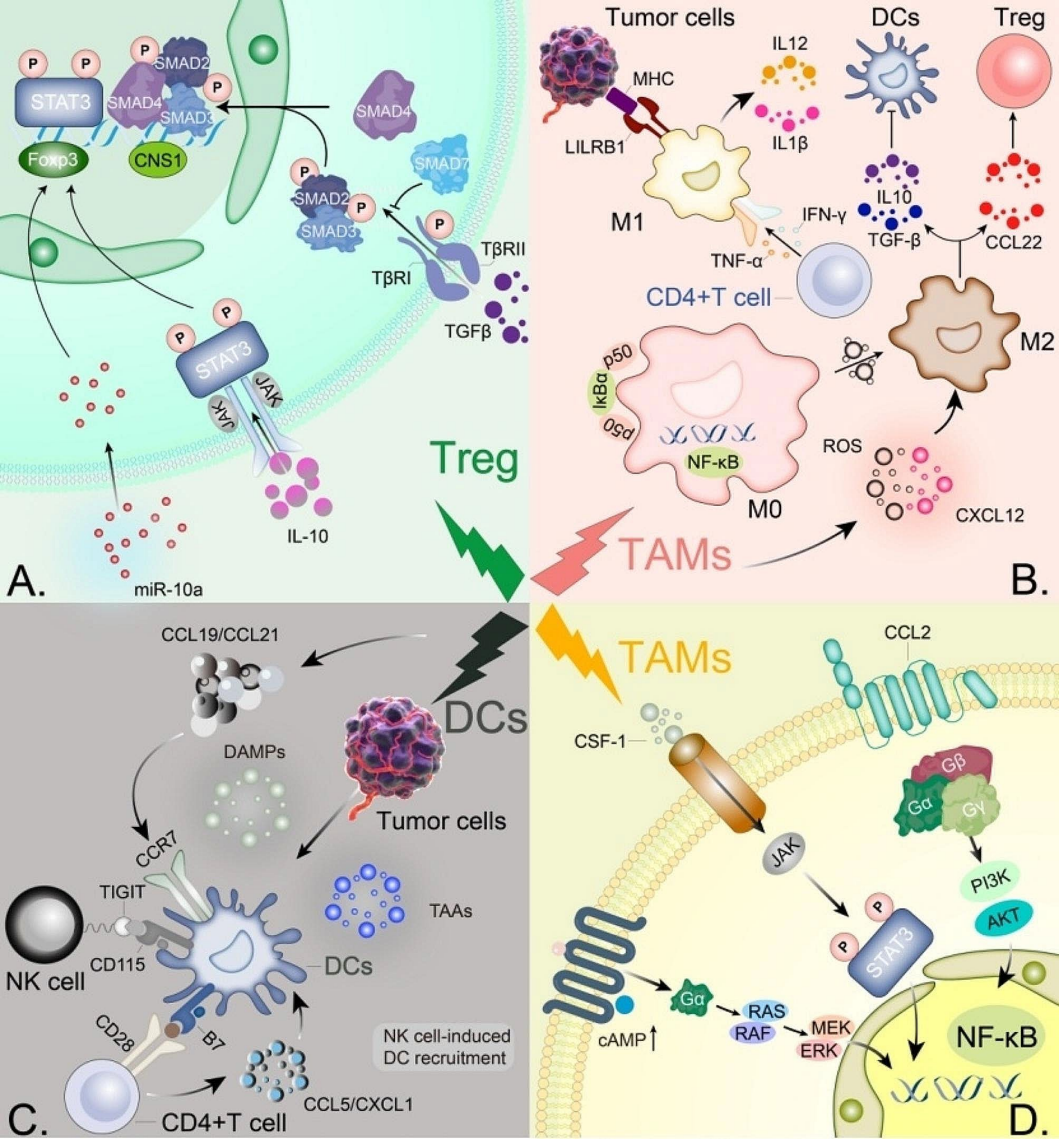
Radiation therapy regulates immune cells and the radiation-related signaling pathways in tumor-associated macrophages (TAMs). (**A**) Radiation enhances the secretion of IL-10, promoting the conversion of CD4 + T cells into Treg cells and enhancing Treg function through the IL-10R-mediated STAT3 signaling pathway. This interaction also activates JAKs, leading to the phosphorylation of STAT3 at the Tyr705 site and subsequent translocation of activated STAT3 dimers into the nucleus. As a co-transcription factor with FOXP3, STAT3 increases the expression of CTLA-4 in Treg cells. Additionally, radiation-induced miR-10a elevates the expression level of FOXP3. Furthermore, radiation significantly increases the levels of TGF-β in the tumor microenvironment (TME). TGF-β recognizes and binds to TGFβRII, leading to the phosphorylation of TGFβRI. TGF-β activates Smad2 and Smad3, promoting the formation of heterotrimeric complexes with Smad4. Smads are recruited to the CNS1 region of the Coxp3 gene locus. CNS1 facilitates the generation, expansion, differentiation, and development of Treg cells. (**B**) Under the influence of radiation-induced p50-p50 NFκB homodimers, M2 macrophages acquire their phenotypes. Moreover, radiation-induced ROS increase further promotes the polarization of M2 macrophages. Activation of p50-p50 dimers facilitates the conversion to the M2 phenotype, resulting in the secretion of IL-10 and TGF-β, which suppresses dendritic cells. The secretion of CCL22 by M2 macrophages also recruits Treg cells to exert immune inhibitory functions. Furthermore, CD4 + T cells activate M1 macrophages through TNF and IFN-γ, enabling them to kill tumor cells through phagocytosis, playing a crucial role in the abstract action. (**C**) Radiation significantly upregulates the expression of CCL19 and CCL21, mediating the migration of dendritic cells. Additionally, radiation-induced immunogenic cell death may release a large amount of antigens and DAMPs, which bind to specific pattern recognition receptors on dendritic cells. Radiation also leads to the release of tumor-associated antigens (TAAs), thereby promoting the activation of dendritic cells, T cell migration, and proliferation. (**D**) Radiation increases the level of CXCL12, which then binds to CXCR4. Similarly, CCL2 and its receptor CCR2 are also activated by radiation. Subsequently, the receptors undergo a conformational change for the second time, activating intracellular trimeric G proteins by dissociating the Gα subunit from the Gβ/Gγ dimer. Phosphoinositide 3-kinases (PI3Ks) are then activated. PI3Ks regulate TAM function through phosphorylation of AKT. Additionally, the Gα subunit activates the Ras and Rac/Rho pathways, leading to ERK phosphorylation. Activated ERK can phosphorylate and regulate other cellular proteins, translocate into the nucleus, phosphorylate and modulate transcription factors, thereby influencing TAM migration, proliferation, and cytokine expression. The Gβ/Gγ dimer also activates the JAK/STAT signaling pathway, promoting changes in cellular morphology and chemotaxis. It is noteworthy that CSF-1/CSF-1R can simultaneously activate all three of the above signaling pathways

On the other hand, radiotherapy can also enhance the anti-tumor effect by increasing T cell activity [54, 124]. Radiotherapy can increase T cell activity, including cytokine production and proliferation, which may enhance the efficacy of cancer treatment [125]. Numerous studies have shown that radiotherapy can lead to increased Treg cell infiltration through a variety of mechanisms, thereby reducing the effectiveness of tumor immunotherapy [126]. Although it is a well-established fact that RT causes Treg cell infiltration, the underlying mechanism remains undiscovered. Firstly, radiotherapy can induce tumor cell death and release a series of cell factors such as HMGB1 and ATP, which can attract and activate Treg cells [127]. Radiation has been shown to upregulate the expression of FOXP3, thereby promoting the proliferation and differentiation of Tregs, and enhancing their function via the STAT3 signaling pathway. Furthermore, radiation can significantly increase the level of TGF-β in the TME, thereby promoting the formation of the heterotrimeric complex Smad2/3/4. This complex activates the CNS1 region at the Foxp3 gene locus, thereby expediting the engendering and maturation of Tregs. Secondly, radiotherapy can destroy tumor cells and release a large amount of antigens, activating the immune system, but at the same time, it can also activate Treg cells and suppress the activity of CD8 + T cells, thereby reducing the immune system’s attack on tumor [128]. Radiation also enhances the production of IL-10 and activates JAK, thereby increasing the nuclear translocation of STAT3 dimers. As a co-transcription factor with FOXP3, STAT3 promotes the proliferation, differentiation, and immune suppression of T cells, and elevates CTLA-4 expression in Tregs. Radiotherapy can cause tumor cells to release a large amount of reactive oxygen species and nitrites, leading to oxidative stress reactions around the tumor and causing inflammation, which activates Treg cells [116] (Fig. 2A).

It is worth mentioning that the clinical efficacy of focal radiotherapy is also affected by the presence of tumor infiltrating cytotoxicity CD8 + T cells. In 2009, Lee and colleagues demonstrated that the radiation effect is primarily mediated by T cells, with cytotoxic CD8 + T cells playing a crucial role, as the tumors exhibited radioresistance following the depletion of CD8 + cells^129^. Single doses of 20 Gy in wild-type mice resulted in substantial tumor regression and an enhanced presence of infiltrating T cells within the tumor microenvironment. Conversely, in T cell-deficient nude mice, the tumors developed radioresistance and underwent progressive growth [129]. This phenomenon was replicated by Takeshima and colleagues, who observed that the depletion of CD8 + T cells led to the elimination of radiation-induced tumor growth inhibition [130]. Numerous analogous experimental studies in mice substantiate the notion that the therapeutic efficacy of irradiation is contingent on the presence of cytotoxic CD8 + T cells. Their experimental depletion significantly and often entirely negated the effects of radiotherapy at dosages of 2 Gy [130], 15 Gy [130], 20 Gy [129], 30 Gy [131–133], 2 × 12 Gy [134], or 5 × 6 Gy [135]. Mice devoid of adaptive immune cells, as well as those depleted of CD4 + or CD8 + T cells, do not exhibit remissions following radiation treatment [136]. Experimental local tumor irradiation significantly elevated levels of tumor-specific CD8 + cytotoxic T cells, demonstrating a close correlation between radiation-induced tumor-specific CD8 + T cells at local tumor sites and radiation-induced tumor growth inhibition [134, 137, 138]. Shortly after a single focal radiation dose of 30 Gy, cytotoxic CD8 + T cells infiltrated subcutaneously implanted murine colon tumors, inducing durable remissions. This effect necessitated the presence of CD8 + cross-priming dendritic cells, while the depletion of either CD8 + T cells or CD4 + T cells significantly diminished survival [133].

#### Tumor-associated macrophages (TAMs)

There are two main subgroups of infantile macrophages (M0): M1 and M2 [139]. The M1 phenotype is recognized as pro-inflammatory cells in conjunction with pro-inflammatory cytokines such as tumor necrosis factor alpha (TNF-α), interferon-γ (IFN-γ), and interleukin 12 (IL-12). In contrast, the M2 phenotype is recognized as anti-inflammatory cells effect in conjunction with anti-inflammatory cytokines such as IL-10, TGF, and IL-6. Notably, TMEs tend to promote the maturation of TAMs toward M2 phenotypes, hence TAMs are commonly referred to as M2 macrophages [140].

TAMs can generate various bioactive molecules that affect tumor growth, metastasis, and treatment response [141–143]. Radiation therapy exerts diverse effects on the functionality and abundance of TAMs [108, 144, 145](Fig. 2B). Parker et al.‘s study indicates that radiotherapy can reduce the differentiation of bone marrow-derived cells, inhibiting the accumulation of TAMs [146]. On one hand, radiotherapy can promote the polarization of M1 macrophages while suppressing that of M2 macrophages [145, 147, 148]. On the other hand, it can influence the expression of secretory factors. Specifically, radiotherapy can decrease the production of immunosuppressive factors by TAMs and simultaneously increase the production of immunostimulatory factors [149, 150]. These changes provide a theoretical basis for sensitizing immunotherapy. Alves R et al.‘s research demonstrates that radiotherapy can reduce the production of immunosuppressive factors, such as IL-10 and TGF-β, by TAMs, while increasing the production of immunostimulatory factors, such as IL-12 and TNF-α [151]. Similarly, Chen et al.‘s study indicates that radiotherapy can enhance the immune response of tumors by suppressing the expression of PD-L1 by TAMs [152]. The NF-κB pathway plays a crucial role in the polarization and expression of secretory factors by TAMs [153, 154]. Studies have shown that radiotherapy can reduce the production of immunosuppressive factors, such as IL-10 and TGF-β, by TAMs by inhibiting the activation of the NF-κB pathway [89, 155].

Radiotherapy can also impact the expression and activity of TAM-related receptors, thereby affecting their function. For instance, Akkari et al.‘s research shows that radiotherapy can decrease the quantity and function of TAMs by inhibiting the activation of the CSF-1R signaling pathway [156]. Likewise radiotherapy can enhance the phagocytic capacity of TAMs towards tumor cells through CD47 inhibition [157](Fig. 2D).

#### Myeloid-derived suppressor cells (MDSCs)

MDSCs play a crucial role in protecting tumors and contributing to their progression [158]. Research has indicated that radiotherapy at the primary tumor site can increase the abundance of MDSCs in certain organs or tissues of the human body [159, 160]. However, in mice with colon tumors, a significant decrease in MDSCs was observed two weeks after a single high-dose radiotherapy treatment [133, 161]. The variability in tumor models, grading, irradiation sites, radiation doses, and analysis timing may account for such differences. MDSCs in the context of RT is regulated by the CSF1/CSF1R signaling pathway, which can limit the efficacy of RT. Blocking CSF1/CSF1R can significantly improve the survival of patients after radiotherapy. Activation of the CSF1/CSF1R signaling pathway is crucial for the recruitment of MDSCs under the context of radiotherapy, which may lead to RT resistance in cancer patients [152, 162]. Further studies indicate that Radiation-induced DNA damage triggers an upregulation of the kinase ABL1, resulting in overexpression of CSF1 in tumor tissue. Furthermore, as reported by Liang et al., the immunosuppressive effect of MDSCs may lead to acquired resistance after local ablative radiation. The STING/I type interferon signaling pathway can recruit MDSCs and enhance suppressive inflammation in tumors [163]. Although the STING pathway has the capacity to reprogram MDSCs into immunostimulatory cells, most studies indicate that it suppresses the function of MDSCs in the TME [164]. The activation of the STING signal mediated by CGAMP enhances the secretion of IFN-γ, thereby inhibiting the generation of MDSCs, indicating the suppressive function of RT on MDSCs [165]. Nonetheless, the complex effects of cGAS/STING signaling on MDSCs need further exploration in the context of RT. SIRT1 has been shown to orchestrate HIF-1α-dependent glycolysis and deregulate function and differentiation of MDSCs when silenced [165, 166]. In summary, RT typically promotes the aggregation and activation of MDSCs, thereby impeding the immune system’s ability to kill cancer cells (Table 1).

#### DCs

DCs are crucial in linking innate and adaptive immunity because of their unique dendritic morphology [167–171]. While PAMPs or “danger signals” are the primary drivers of DCs activation through respective receptors, radiation-induced DCs activation is predominantly mediated by DAMPs such as HMGB1 and calreticulin (Fig. 2C). After tumor RT, CD70 and CD86 co-stimulatory molecules on DCs are upregulated [172]. It is known that radiation can enhance the expression of the priming factor and major histocompatibility complex(MHC) I [173–175]. Radiation-induced immunogenic cell death releases antigens and DAMPs, including HMGB1, ATP, calreticulin, and HSPs, which activate DCs through various receptors (such as TLRs and NOD-like) [176]. Calreticulin, which is upregulated by radiation, acts as a pro-phagocytosis “eat-me” signal, while HMGB1 released from tumor cells promotes antigen presentation by DCs via TLR4 activation [177].

Research indicates the existence of immune crosstalk mediated by DCs between non-neoplastic primary foci, thereby highlighting profound interest in the immunomodulatory cross-regulation between the intestinal and pulmonary domains [178–181].

**Fig. 3:**
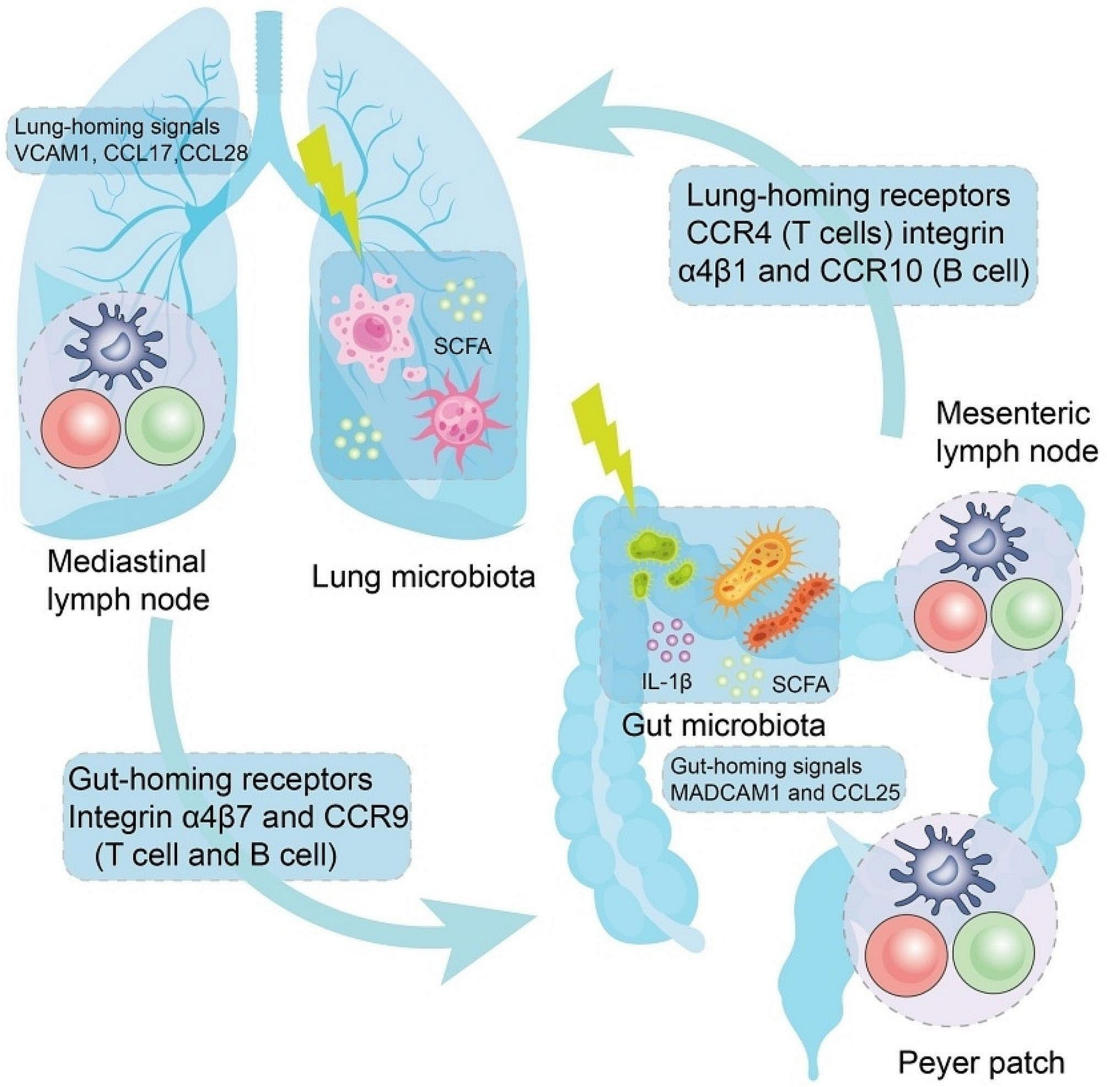
Putative homing signals governing the intricate immune interplay between the mucosal domains of the lungs and gastrointestinal tract. Dendritic cells (DCs) residing in lymph nodes that drain specific tissues possess the ability to imprint lymphocytes with specialized cues, facilitating their return to the corresponding tissue. It is postulated that mucosal sites establish a network wherein migratory interactions can take place. Reports indicate the intriguing possibility of reciprocal imprinting of well-known homing signals, thereby redirecting the movement of immune cells across various organs. This phenomenon may underlie the observed immune responses elicited at distant sites beyond the area subjected to radiotherapy (RT). Moreover, RT has demonstrated its influence on both local and distant variations in the composition of microbiota communities. Such alterations might arise from the circulation of microbiota-derived substances, such as short-chain fatty acids (SCFAs) or IL-1β, traversing the mucosal system between the gastrointestinal tract and the lungs. As of now, the comprehensive mechanism governing these homing signals and the intricate interplay between the gut and lungs, referred to as gut-lung crosstalk, remains shrouded in mystery. CCL, denoting CC-chemokine ligand, plays a significant role in this context

Highly specific tissue homing signals play a crucial role in RT-induced immune crosstalk. Specifically, antigen-carrying DCs are recruited to locally draining lymphoid structures. Bronchus-associated lymphoid tissue (BALT) serves as a vital component of mucosa-associated lymphoid tissue. Peyer patches and mesenteric lymph nodes represent the principal constituents of gut-associated lymphoid tissue, acting as bridging elements for immune crosstalk between the intestines and lungs [182]. Within the context of gut-associated lymphoid tissue, DCs have the ability to upregulate the expression of integrinα4β7 and CCR9, and selectively interact with MADCAM1 and CCL25, thereby imprinting T cells and B cells (Fig. 3) [183]. Furthermore, DCs migrating from the lungs to BALT can imprint T cells with the expression of CCR4, facilitating binding with CCL1 expressed by pulmonary epithelial cells. Activation of B cells in DC-mediated BALT results in the upregulation of integrin α4β1 and CCR10. In colonic and pulmonary tissues, these molecules promote cellular migration through interactions with VCAM1 and CCL28 [184]. Consequently, dendritic cells are capable of orchestrating the homing of T cells and B cells to local organs [183]. It is worth noting that DC-mediated pulmonary imprinting can facilitate cellular migration towards the intestines. In line with this, DCs originating from the lungs demonstrated the ability to imprint the expression of gut-homing molecules, namely integrin α4β7 and CCR9, on T cells that were co-cultured in vitro and on cells transferred into living organisms. Moreover, in a murine model of respiratory infection caused by influenza virus, lung-derived CCR9 + T cells played a pivotal role in mediating intestinal immune injury by homing to the intestine [185]. Subsequently, within the intestinal milieu, T cells producing IFNγ induced alterations in the intestinal microbiota, leading to an inflammatory response mediated by TH17 cells [186]. These examples illustrate the plasticity of tissue-specific homing signals and offer a potential explanation for the observed radiation therapy-induced immune effects in distal sites beyond the irradiated field.

## iRT enhcances abscopal effect

RT is a well-established therapeutic modality for various types of cancer. However, its clinical utility has been limited by the development of resistance and the inability to target distant metastases. Recently, immunotherapy, which leverages the power of the immune system to fight cancer, has shown promising results in the treatment of advanced malignancies [187–194]. The combination of RT and immunotherapy has emerged as a potential strategy to enhance the abscopal effect of radiotherapy and improve clinical outcomes. RT activates the immune system to recognize and attack cancer cells. One mechanism of this combination is the release of tumor-associated antigens (TAAs) by irradiated tumor cells. These antigens can then be presented to immune cells, such as T cells, which recognize and attack cancer cells expressing these antigens. The release of TAAs is not the only mechanism by which RT can enhance the abscopal effect of immunotherapy [195, 196]. RT also induces immunogenic cell death (ICD), a type of cell death that triggers an immune response [197, 198]. The process can lead to the release of specific antigens from tumor cells, thereby stimulating the clonal expansion of tumor-specific T lymphocyte subsets [199, 200]. Antigen-presenting cells (APCs) capture specific antigens and combine them with the MHC, then present the antigens to activate T helpe cells. The activated T cells mainly include cytotoxic T lymphocytes and NK cells, which can exert anti-tumor immune effects and effectively eliminate tumor cells. Therefore, the key mechanism of radiotherapy-induced ICD lies in stimulating the recruitment and differentiation of T lymphocytes, promoting the recognition and effective attack of tumor cells by T lymphocytes [201]. Research suggests that radiotherapy can induce the formation of ROS and other oxidative stress sources, triggering endoplasmic reticulum stress to mediate tumor ICD [202]. Li et al. found that near-infrared irradiation could induce a strong endoplasmic reticulum stress response and calreticulin exposure on the cell surface, thereby stimulating the antigen-presenting function of DCs [203]. This, in turn, promotes ICD-related anti-tumor immune responses through the activation of various immune responses, such as increased proliferation of CD8 + T lymphocytes and secretion of cytotoxic cytokines. Therefore, the ICD process involves the release of various antigens and DAMPs, which participate in the activation of immune response signaling pathways and promote anti-tumor immune responses [204]. DAMPs are key molecular steps necessary for radiotherapy-induced ICD, including the surface expression of calreticulin and heat shock proteins, the release of high mobility group protein B1, and the active secretion of adenosine triphosphate ATP [205]. DAMPs can increase the expression of TAAs, which are mainly neoantigens caused by immunogenic mutations driven by radiotherapy [206]. In addition to the aforementioned effects, RT can also enhance the infiltration of immune cells into the TME, which is of paramount importance since tumors usually create an immunosuppressive milieu that hinders immune cells from recognizing and attacking cancer cells [207, 208]. RT can disrupt this immunosuppressive environment by inducing the expression of chemokines, such as CXCL16, which attract immune cells to the tumor site [209]. Moreover, RT can upregulate adhesion molecules, such as ICAM-1, which facilitate the adherence of immune cells to the endothelium and their subsequent migration into the TME [210, 211]. Another avenue through which RT boosts the abscopal effect of immunotherapy is by altering the expression of immune checkpoint molecules [3, 4, 212]. Immune checkpoint molecules are surface proteins on immune cells that regulate immune responses. Malignant cells can manipulate these molecules to evade detection and elimination by the immune system. However, RT can stimulate the overexpression of checkpoint molecules, such as PD-L1, which can be targeted by ICIs. This can augment the effectiveness of ICIs by activating immune cells, leading to improved clinical outcomes.

The combination of radiotherapy and immunotherapy has demonstrated significant effectiveness in treating various types of cancers. A study has shown that metastatic melanoma patients who received a combination of radiotherapy and immune checkpoint inhibitor, ipilimumab, had an improved overall survival compared to those who received radiotherapy or ipilimumab alone [213]. In another study, non-small cell lung cancer patients who received a combination of radiotherapy and another immune checkpoint inhibitor, pembrolizumab, showed an improved progression-free survival compared to those who received radiotherapy alone or pembrolizumab alone [24]. In summary, the combination of radiotherapy and immunotherapy holds great potential in augmenting the abscopal effect of radiotherapy by activating the immune system and modulating the TME, leading to a systemic immune response and regression of distant tumors. While preclinical studies have provided promising results, further research is required to optimize the combination therapy and evaluate its efficacy in clinical settings. Therefore, our next focus will be on the current clinical applications in radiotherapy combined with immunotherapy.

## Clinical practice

The immunomodulatory effects of RT have opened up a new avenue for cancer treatment. The potential of combining RT with immunotherapy has been widely explored in recent years, with significant success in treating various cancers, such as NSCLC, melanoma, and solid tumors [214–219]. Several clinical trials have demonstrated the effectiveness of iRT in achieving local control of lesion sites and inducing an abscopal effect, which is the regression of non-irradiated metastatic lesions. This novel approach represents a major breakthrough in cancer therapy. We will elucidate the salient aspects and latest developments of clinical practice in iRT through the following four facets.

### Summary of clinical trials

Owing to the substantial immunomodulatory effects of RT, its application can be extensively utilized in the form of iRT. Indeed, iRT has proven remarkably effective on various cancers, including non-small cell lung carcinoma (NSCLC), melanoma, and certain solid tumors [215, 220, 221]. In 2015, the first attempt at iRT was initiated, and researchers demonstrated a significant abscopal effect in patients with metastatic solid tumors through the combination of radiotherapy and GM-CSF. This trial provided a foundation for subsequent research and clinical trials on iRT, which have yielded promising results [47]. Based on the findings from KEYNOTE-001 regarding the efficacy and safety of pembrolizumab, Shaverdian et al. further explored that patients with advanced NSCLC who had previously undergone radiotherapy experienced extended PFS and OS with pembrolizumab compared to those without prior radiotherapy, maintaining an acceptable safety profile. This study was the most comprehensive at the time to document the influence of prior radiotherapy on the efficacy and toxicity of ICIs [222]. Beyond PD-1/PD-L1 inhibitors, the synergy between radiotherapy and CTLA-4 inhibitors has also demonstrated both abscopal and therapeutic effects. Formenti et al. discovered that patients with NSCLC who were previously unresponsive to IO had a positive response after treatment with a combination of RT and CTLA-4 blockade, leading to significantly extended survival time in some cases [23]. The most compelling evidence advocating for improved iRT was observed in the phase III PACIFIC trial, which disclosed that individuals suffering from locally advanced, unresectable NSCLC demonstrated a median PFS of 16.8 months when treated with the PD-L1 antibody durvalumab, in contrast to 5.6 months in patients receiving a placebo [223]. These findings demonstrate the potential of iRT in the treatment of NSCLC and highlight the need for further clinical trials to investigate the efficacy and safety of this approach in other types of cancer [224–226]. Moreover, patient-reported outcomes and long-term survival rates in the study confirmed the clinical benefit of IO combined with chemoradiotherapy [24, 227]. Based on Pembro-RT trial, a pooled analysis of two randomized trials from the Netherlands and MD Anderson including Pembro-RT showed that the effect of RT combined was statistically valid in advanced NSCLC [227, 228]. However, the choice of ICIs and RT including agents, sequence, dose, fractionation, and irradiated sites to exert the best synergies needs to be explored and optimized [229]. Hereafter, we attempt to summarize the existing clinical trials to look at the possible optimal combination of iRT and the broad prospect of iRT to treat locally advanced and advanced tumors. However, the optimal combination of ICIs and RT, including the agents, sequence, dose, fractionation, and irradiated sites, needs to be further explored and optimized [229]. Therefore, a systematic review of existing clinical trials is necessary to identify the most effective iRT approach for locally advanced and advanced tumors.

In view of the results of current completed or partially completed clinical trials, the combination mode, sequence and radiotherapy dose selection of radiotherapy and immunotherapy combined application are great challenges. On the other hand, radiotherapy can greatly change the tumor immune microenvironment. Based on the immune response mechanism triggered by radiotherapy, it provides theoretical basis and targets for the development of immunomodulators.

### Selection of radiation dose

Despite the established potential of iRT, there remain important considerations to address in its clinical use [230–233]. One such consideration is determining the optimal dose of radiation for achieving the desired immune response. As discussed previously, the immune response to radiation is dependent on the dose delivered, and thus the optimal dose for iRT should maximize tumor immunity while remaining tolerable for patients. A preclinical study using mice bearing B16-ovalbumin murine melanoma found that a dose of 7.5 Gy per fraction of radiation may achieve better outcomes while maintaining low numbers of Tregs, compared to a dose of 5 Gy per fraction. This highlights the importance of carefully considering radiation dose in iRT treatment planning. However, further research is needed to determine the optimal dose for iRT in humans [234]. Another study demonstrated that a single dose of 15 Gy of radiation led to an increased infiltration of host immune cells into tumors when compared to a fractionated schedule of 3 Gy X 5 [173]. However, the use of high-dose radiation (≥ 15 Gy) may result in a higher proportion of splenic Tregs, which could suppress the antitumor immune response [235]. Some preclinical studies have suggested that conventional fractionation may be more effective when combined with IO [236]. In certain cancers, hypofractionated RT has shown promise in clinical practice. Combining IO with SBRT may also have potential in the modality of iRT [237]. A subgroup analysis of the PACIFIC study has demonstrated that patients can achieve a significant survival benefit regardless of the radiation dose used [238]. The published content is discussing the results of a randomized phase I/II trial that investigated the effects of combining immunotherapy with different types of radiotherapy for treating lung and liver lesions of NSCLC [24]. The trial included 382 participants, and the results showed that the combination of pembrolizumab and hypofractionated radiotherapy (50 Gy in 4 fractions) had better out-of-field ORRs and longer median PFS times compared to the combination of pembrolizumab and traditional radiotherapy. These findings suggest that hypofractionated radiotherapy may enhance the effectiveness of immunotherapy [239]. However, according to another study, the combination of SBRT with PD-1 inhibitors and low-dose cyclophosphamide did not confer benefits compared to conventional radiotherapy in patients with metastatic colorectal cancer [240]. It is possible that the observed differences in clinical outcomes are due to the variations in tumor types and their heterogeneity. Despite these findings, the results of the trial suggest that both SBRT and hypofractionated RT may enhance the immune response against tumors, but the optimal dose for patients still requires further investigation through additional clinical studies. Notably, there are a large number of ongoing clinical trials in this area (Table 2), which may provide valuable data for the selection of RT modalities.

**Table 1:** Immunomodulatory factors and their functions in TME induced by radiation

Immune Regulatory Factor	Role in TME	Radiotherapy-Induced Changes
Antigen-Presenting Cells (APCs)	Present tumor antigens to T-cells	Increases antigen presentation and activation of APCs
Cytokines	Regulate immune response	Increases production of pro-inflammatory cytokines, such as IL−6 and TNF-α, while decreasing production of anti-inflammatory cytokines, such as TGF-β
Chemokines	Attract immune cells to tumor site	Increases expression of chemokines, such as CXCL9 and CXCL10, which attract T-cells and natural killer cells
Danger-Associated Molecular Patterns (DAMPs)	Activate immune response against cancer cells	Increases release of DAMPs, such as HMGB1, which activate immune response
Pathogen-Associated Molecular Patterns (PAMPs)	Activate immune response against cancer cells	Increases release of PAMPs, such as dsRNA, which activate immune response
Immune Checkpoint Molecules	Regulate immune response	Modulates expression of immune checkpoint molecules, such as PD−1, CTLA−4, and TIM−3, which can lead to increased T-cell activation and decreased immune suppression
Regulatory T-cells (Tregs)	Suppress immune response	Decreases Treg activity and promotes a pro-inflammatory environment
Myeloid-Derived Suppressor Cells (MDSCs)	Suppress immune response	Decreases MDSC activity and promotes a pro-inflammatory environment

**Table 2:** Representative trials using combination of radiotherapy and immunotherapy

Clinical Trial	Condition or disease	Radiotherapy Dose	Immunotherapy	Sequence	OS/PFS	Results
NCT02125461	NSCLC	54 to 66 Gy	Durvalumab	RT, IO	4 years/3 years	Durable PFS and sustained OS benefit with durvalumab after chemoradiotherapy
NCT02608385	Solid tumors	SBRT dosing varied by site and ranged from 30 to 50 Gy in three to five fractions	Pembrolizumab	SBRT, IO	24 months/12 months	Well tolerated with acceptable toxicity
NCT02434081	NSCLC	66 Gy in 33 fractions	Nivolumab	Concurrent	4 years/3 years	The addition of nivolumab to concurrent CRT is safe and tolerable
NCT02444741	NSCLC	Various	Pembrolizumab	Concurrent	5 years/3 months	Safe and more beneficial for patients with low PD-L1 expression
NCT02343952	Carcinoma, NSCLC	59.4 to 66.6 Gy	Pembrolizumab	RT, IO	47 months/47 months	PFS and OS improvement with consolidation pembrolizumab
NCT03631784	NSCLC	60 Gy in 30 daily fractions	Pembrolizumab	Concurrent	5 years/5 years	Promising antitumor activity and manageable safety
NCT03035890	Metastatic NSCLC	Hypo-fractionated Radiation	Immuno-Therapeutic Agent (Nivolumab/ pembrolizumab/ atezolizumab)	Concurrent	2 years/2 years	Active, not recruiting
NCT05111197	Locally advanced or metastatic NSCLC	SBRT	Anti-PD−1 or anti-PD L1 immunotherapy	IO, RT	12 months/12 months	Not yet recruiting
NCT03523702	Locally Advanced NSCLC	Selective personalized radiotherapy	Pembrolizumab	Concurrent	18 months/18 months	Recruiting
NCT03383302	NSCLC Stage II and Stage I	SBRT	Nivolumab	RT, IO	6, 12 and 24 months/-	Recruiting
NCT04577638	NSCLC Stage III	Intensity Modulated Radiotherapy	Nivolumab	Concurrent	-/-	Recruiting
NCT03168464	NSCLC Metastatic	6 Gy x 5 fractions	Ipilimumab/ nivolumab	Concurrent	4 years/4 years	Recruiting
NCT03110978	Stage I-IIA or Recurrent NSCLC	SBRT	Nivolumab	Concurrent	5 years/-	Recruiting
NCT03313804	NSCLC, Squamous Cell Carcinoma of the Head and Neck	SBRT	Nivolumab/ pembrolizumab/ atezolizumab	IO, RT	-/-	Recruiting
NCT05229614	NSCLC, Head and Neck Squamous Cell Carcinoma, Melanoma, Urothelial Carcinoma	Carbon ion therapy	Pembrolizumab	IO, RT	8 weeks/8 weeks	Not yet recruiting
NCT05265650	Metastatic NSCLC	SBRT	Nivolumab	Concurrent	-/ 1 year	Not yet recruiting
NCT05222087	Metastatic NSCLC	SBRT	Pembrolizumab	RT, IO	-/24 months	Not yet recruiting

### Selection of immunotherapy modality

To enhance antitumor immunity, hypofractionated RT has shown promising results. However, it remains unclear which ICIs is most effective when combined with RT. Several studies have demonstrated the efficacy of RT in combination with ICIs, such as pembrolizumab and atezolizumab [223, 241–243]. This suggests that using a combination of RT and an ICI could potentially improve treatment outcomes. Based on the most commonly used ICIs, PD-1 and CTLA-4 blockades, a retrospective analysis was conducted on two single-institution prospective trials. The study found that the failure progression survival of anti-PD1 combined with stereotactic body radiation therapy (SBRT) for metastatic NSCLC was significantly superior to anti-CTLA4 combined with SBRT. However, the study was unable to determine which combination was superior. This suggests that anti-PD1 combined with SBRT may be a more effective treatment option for metastatic NSCLC patients compared to anti-CTLA4 combined with SBRT [215]. The preliminary findings from this study suggest that the anti-CTLA4 treatment had a lower PFS rate compared to anti-PD1 treatment in patients with metastatic NSCLC. Specifically, the PFS was 76% and 94% at 3 months, 52% and 87% at 6 months, 31% and 80% at 12 months, and 23% and 63% at 18 months, respectively (*p* = 0.02). However, it is important to note that this is a preliminary exploration and further data is required to confirm these findings. Furthermore, the results are limited to metastatic NSCLC and additional studies are needed to evaluate the effectiveness of ICIs in other types of tumors. It is also important to consider the use of one or multiple ICIs in the context of multi-site RT. Additional clinical trials are necessary to further investigate and expand upon the findings in this area.

### Ideal timing for iRT

Combining RT and immunotherapy is an important treatment strategy for cancer, with the optimal timing being a crucial factor. In addition to the selection of therapy modality, it is generally observed that either concurrent or sequential administration of checkpoint inhibitors after RT can be effective [230–232]. The PACIFIC trial is a well-known example of the success of combining chemoradiotherapy with immunotherapy. The trial established the framework for adjuvant administration of durvalumab after chemoradiotherapy for NSCLC patients in stage III [223, 226]. Durvalumab administration improved PFS and estimated 4-year OS rates significantly, with 4-year PFS rates of 35.3% versus 19.5% for placebo. Additional clinical trials have corroborated the safety and effectiveness of pembrolizumab and nivolumab following chemoradiotherapy [241, 244]. Nonetheless, a recent retrospective cohort analysis revealed that 81.8% of patients treated with durvalumab subsequent to concurrent chemoradiotherapy developed pneumonitis, with 59.5% remaining asymptomatic. This study highlights the importance of carefully monitoring patients for potential side effects when combining these treatments. Overall, combining RT and immunotherapy can be an effective treatment strategy for cancer, but the optimal timing and careful patient monitoring are crucial factors in ensuring its success. A study conducted in a real-world setting on the use of durvalumab consolidation therapy following chemoradiotherapy in stage III NSCLC patients revealed that the incidence of grade 3 radiation pneumonitis was significantly higher in the durvalumab group (14.3%) compared to the observation group (2.5%) [245]. This highlights the need to address adverse effects and improve the PACIFIC mode. Analysis of clinical data, including the PACIFIC study, suggests that IO given concurrently or soon after RT improves patient outcomes compared to IO started later. The updated data from the PACIFIC study shows significant improvement in OS and FPS both within and after 14 days of combining IO with RT [226]. However, a retrospective study has demonstrated that patients who received IO 21 days or more after the onset of SBRT experienced longer OS than those who received IO within 21 days. The study conducted has numerous confounding factors, indicating the necessity to further explore the optimal timing of iRT through large randomized clinical trials.

### Toxicity of iRT

Despite the breakthrough achieved by iRT in preclinical and clinical trials, the potential toxicity of this combination strategy remains a significant concern. The side effects of RT are local or locoregional, affecting those tissues or organs that have been exposed to radiation [246, 247]. “Early side effects” are those presenting within weeks or months of treatment completion, while “late side effects” occur months or years after treatment. It has been shown that patients received by RT suffer from distress, anxiety, and depression. Many patients still experience psychological effects after treatment, even though they tend to decrease over time [248]. Moreover, up to 80% of patients who undergo RT experience acute fatigue after the procedure, and chronic fatigue can persist for months or years afterward [249]. In addition to the systemic symptoms described above, organ-specific adverse reactions occur in patients receiving RT. Often, oral mucositis occurs as a first sign of RT in head-and-neck cancer, characterized by inflammation or ulceration of the oral or oropharyngeal mucosa [250]. Additionally, there can be gastrointestinal toxicity, sexual dysfunction, and concerns regarding fertility in pelvic cancers which are more frequently treated with RT than other cancers [251]. Thoracic radiotherapy can cause radiation-induced lung injury (RILI) which elaborated below or radiation-induced heart disease such as myocardial infarction (MI), valve damage, and congestive heart failure induced by radiation [252, 253].

One of the most serious consequences of radiotherapy is radiation-induced lung injury (RILI) [254]. While radiation pneumonitis typically develops within 6 months following radiotherapy, radiation pulmonary fibrosis can take over a year to appear [255, 256]. The primary molecular mechanism underlying RILI is the activation of the TGF-β signaling pathway. Upon radiation exposure, TGF-β is released from activated macrophages and other cells in the lung, leading to the activation of its downstream signaling pathways, including the Smad and non-Smad pathways. This results in the increased production of extracellular matrix proteins, such as collagen, fibronectin, and elastin, leading to the development of pulmonary fibrosis [257–260]. Other molecular mechanisms involved in the pathogenesis of radiation-induced pulmonary fibrosis include the activation of NF-κB signaling pathway and the upregulation of pro-inflammatory cytokines and chemokines. Upon radiation exposure, NF-κB is activated, leading to the upregulation of pro-inflammatory cytokines and chemokines such as IL-1/6, TNF-α, and MCP-1. These cytokines and chemokines attract inflammatory cells to the lung, leading to the development of pulmonary fibrosis [261, 262]. In addition to these mechanisms, radiation-induced oxidative stress, DNA damage, and inflammation also play a role in the pathogenesis of pulmonary fibrosis. Radiation exposure leads to the production of ROS, which can cause oxidative damage to cellular components, including DNA. This can activate the DNA damage response, leading to the production of pro-inflammatory cytokines and chemokines, further promoting the development of pulmonary fibrosis [263–265].

Understanding the molecular mechanisms involved in the pathogenesis of radiation-induced pulmonary fibrosis is crucial for the development of effective treatment and prevention strategies. Several approaches have been investigated to prevent or treat pulmonary fibrosis, including anti-inflammatory agents, antioxidants, and agents that target the TGF-β pathway [266–268]. One potential approach is the use of TGF-β receptor inhibitors, which block the downstream signaling pathways activated by TGF-β. Several TGF-β receptor inhibitors, including pirfenidone, have been shown to be effective in reducing pulmonary fibrosis in preclinical studies and clinical trials. Pirfenidone is an orally active small molecule that inhibits TGF-β signaling and has been approved for the treatment of idiopathic pulmonary fibrosis. In a study on radiation-induced pulmonary fibrosis in rats, treatment with pirfenidone significantly reduced fibrosis and improved lung function [269]. Another potential approach is the use of antioxidants, which can scavenge ROS and reduce oxidative stress. N-acetylcysteine is an antioxidant that has been investigated for its potential in the prevention and treatment of radiation-induced pulmonary fibrosis. In a study on radiation-induced pulmonary fibrosis in mice, treatment with NAC significantly reduced pulmonary fibrosis and improved lung function [270].

One of the most common side effects of immunotherapy is immune-related adverse events (irAEs). IrAEs are caused by the immune system attacking healthy cells and tissues in the body, leading to inflammation and damage [271–274]. The severity and type of irAEs depend on the type of immunotherapy, the dose, and the patient’s individual response to treatment. Some of the most common irAEs associated with immunotherapy include dermatitis, colitis, pneumonitis, and thyroiditis [275–277]. The molecular mechanism underlying irAEs is not completely understood, but it is thought to involve the activation of immune cells, particularly T cells. In normal circumstances, T cells are responsible for recognizing and attacking foreign cells, such as viruses and bacteria. However, in the context of immunotherapy, T cells can also attack healthy cells and tissues, leading to irAEs [278–280]. One study investigated the role of T cells in the development of irAEs in patients receiving anti-PD-1 therapy for melanoma. The researchers found that patients who developed irAEs had higher levels of activated T cells in their blood compared to patients who did not develop irAEs. The activated T cells were also found to produce higher levels of pro-inflammatory cytokines, which are known to be involved in the development of irAEs. The study suggests that T cells play a crucial role in the development of irAEs and that targeting these cells may be a potential strategy for preventing or treating irAEs [281]. Another study explored the role of gut microbiota in the development of irAEs in patients receiving anti-CTLA-4 therapy for melanoma. The researchers found that patients who developed colitis, a common irAE, had a different composition of gut microbiota compared to patients who did not develop colitis. Specifically, the patients with colitis had a lower abundance of certain bacteria, such as Bacteroides fragilis, which are known to have anti-inflammatory properties. The study suggests that gut microbiota may play a role in the development of irAEs and that manipulating the composition of gut microbiota may be a potential strategy for preventing or treating irAEs [282, 283]. There is currently no evidence to suggest that the toxicity of iRT is significantly higher than the toxicity of using ICIs or RT alone. According to Teng et al., the use of ICIs in areas of the body previously treated with RT can cause an inflammatory response. This response involves the infiltration of lymphocytes and the release of cytokines, potentially explaining the mechanism behind radiation recall pneumonitis [284].

## Nanomaterials trigger abscopal effect in immunotherapy of metastatic cancers

Nanomaterials have emerged as a promising tool in cancer immunotherapy due to their unique properties and ability to modulate the immune system [285–288]. One potential application of nanomaterials in cancer immunotherapy is their ability to trigger abscopal effects [289, 290]. One of the key advantages of nanomaterials is their ability to accumulate at the tumor site through the enhanced permeability and retention effect (EPR). This enables the targeted delivery of therapeutic agents, such as immunomodulators or drugs, to the TME. In addition, nanomaterials can be designed to modulate the immune system, including enhancing the activation and proliferation of immune cells and reducing the immunosuppressive effects of the TME [291–294].

**Fig. 4:**
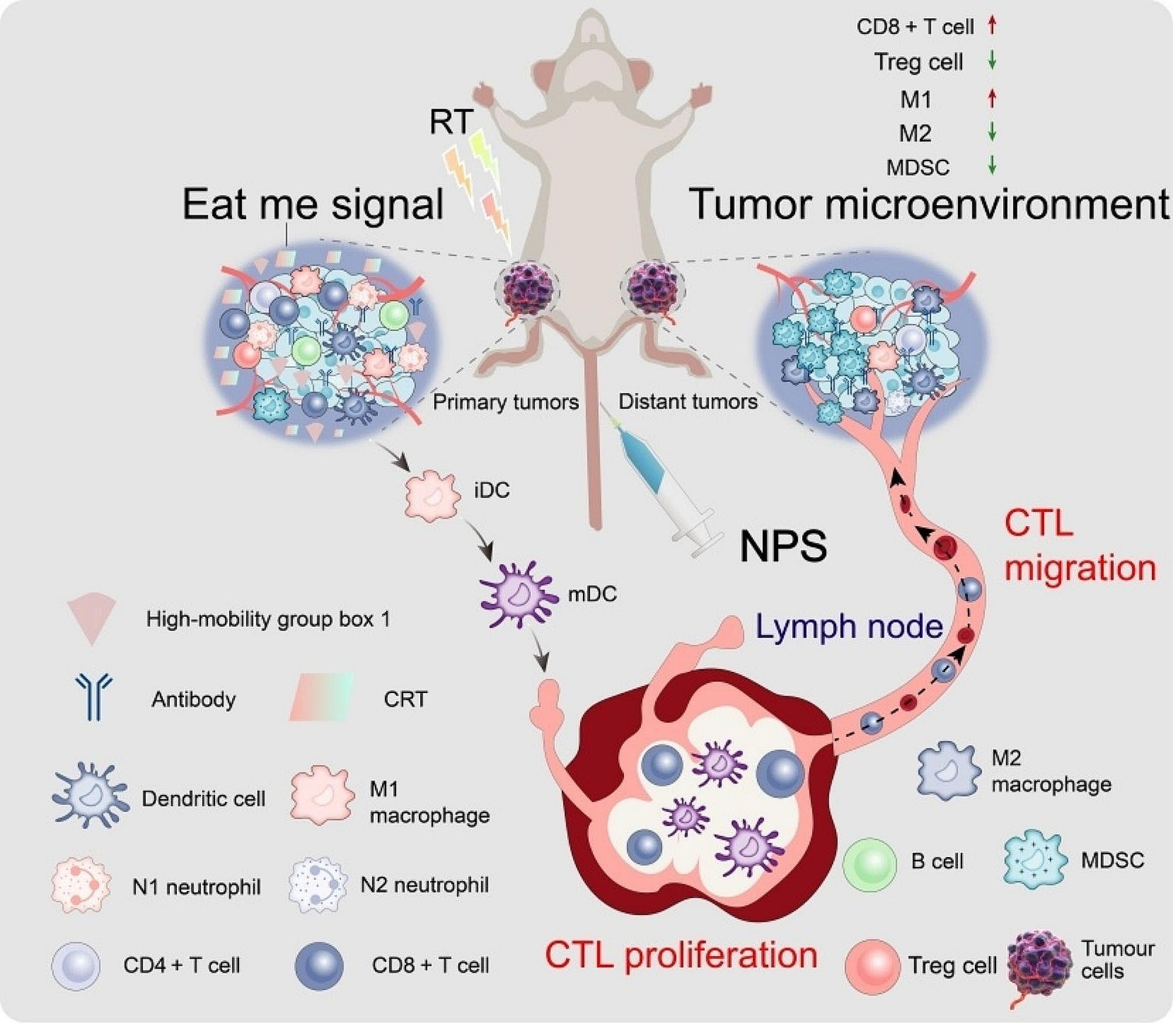
Schematic illustration depicting the role of nanomaterials in eliciting the abscopal effect within the context of immunotherapeutic intervention for metastatic cancer. Nanomaterials instigate the abscopal effect when utilized in conjunction with radiotherapy (RT), leading to a surge in CD8 + T cells and M1 tumor-associated macrophages (TAMs) within the remote TME. Simultaneously, this process engenders a decline in regulatory T cells (Tregs), myeloid-derived suppressor cells (MDSCs), M2-TAMs, and cancer-associated fibroblasts (CAF)

One strategy for using nanomaterials to induce abscopal effects in cancer immunotherapy is to combine them with RT (Fig. 4). RT can induce immunogenic cell death, releasing DAMPs that activate the immune system. However, the abscopal effect is rare and not consistently observed in all patients. Nanomaterials can be used to enhance the immunogenicity of RT and increase the likelihood of triggering an abscopal response [295, 296]. Several studies have demonstrated the potential of nanomaterials to enhance the abscopal effect of RT in cancer immunotherapy. For example, a study by Zheng et al. used gold nanorods to enhance the abscopal effect of RT in a mice model of breast cancer. The gold nanorods were conjugated with an immune adjuvant, CpG oligonucleotides, and injected into the TME. The mice were then treated with RT to one tumor site. The combination of RT and the gold nanorods significantly enhanced the abscopal effect, resulting in regression of tumors at the non-irradiated site [297]. In addition, researchers utilized nanoscale metal-organic frameworks (NMOFs) to augment the abscopal impact of RT in a murine model of melanoma. The NMOFs were impregnated with a TLR7 agonist, which is a variety of immune adjuvant, and subsequently administered into the tumor microenvironment. The mice were then treated with RT to one tumor site. The combination of RT and the NMOFs significantly enhanced the abscopal effect, resulting in regression of tumors at the non-irradiated site [298].

However, while these studies demonstrate the potential of nanomaterials to enhance the abscopal effect of RT, there are still many challenges to overcome (Table 3). One challenge is to ensure the safety and biocompatibility of the nanomaterials used. Some nanoparticles, such as carbon nanotubes, have been found to have toxic effects on cells and tissues, leading to concerns about their use in medical applications. However, many researchers are working to develop safer nanomaterials that can be used in clinical settings. Another challenge is to optimize the design of the nanomaterials to achieve the desired therapeutic effect. The size, shape, surface chemistry, and other properties of nanoparticles can all affect their behavior in the body and their interaction with cells and tissues [292, 299, 300]. Therefore, careful design and optimization of nanomaterials will be necessary to ensure their effectiveness in triggering abscopal effects in immunotherapy.

**Table 3:** Advantages and disadvantages for future research regarding approaches for boosting the abscopal effect

Method	Advantages	Disadvantages
High-dose radiation	- Induces immunogenic cell death - Enhances antigen presentation - Increases T cell infiltration	- Can also cause T cell exhaustion and immune suppression - Risk of toxicity with higher doses
Combination with immunotherapy	- Synergistic effect - Enhances systemic immune response	- Potential for increased immune-related adverse events - Cost and accessibility of immunotherapy
Toll-like receptor agonists	- Enhances dendritic cell maturation and T cell activation	- Limited efficacy in monotherapy - Potential for systemic inflammation
STING agonists	- Activates type 1 interferon pathway and enhances immune response	- Limited data in humans - Potential for systemic inflammation
Targeted therapies	- May induce immunogenic cell death and enhance antigen presentation - Potential synergy with immunotherapy	- Limited data on abscopal response - Potential for immune related adverse events
Gene therapy	- Can introduce genes encoding immune -stimulatory molecules or chimeric antigen receptors (CARs) to enhance immune response	- Limited data on abscopal response - Potential for immune related adverse events - Challenges with delivery and scalability
Nanoparticles	- Can deliver antigens and adjuvants to antigen -presenting cells to enhance immune response	- Limited data on abscopal response - Potential for toxicity with higher doses - Challenges with delivery and scalability
Hyperthermia	- Enhances tumor cell susceptibility to radiation and induces immune response	- Limited data on abscopal response - Potential for systemic inflammation and immune suppression
Exercise	- Enhances immune function and increases T cell infiltration into tumors	- Limited data on abscopal response - Potential for physical limitations and adherence issues

## Future prospective and conclusion

The abscopal effect has shown great promise in cancer treatment. The ability of RT to induce an immune response and target distant tumors has the potential to revolutionize cancer treatment, especially for patients with metastatic disease. Immunotherapy drugs such as checkpoint inhibitors, which block the negative immune regulation, have been shown to enhance the immune response to RT. Clinical trials have shown promising results with the combination of RT and checkpoint inhibitors in treating metastatic melanoma and lung cancer. Moreover, localized RT, such as stereotactic body RT, has the potential to enhance the abscopal effect by delivering high doses of radiation to a specific area while minimizing radiation exposure to healthy tissues. Radiosensitizers are drugs that enhance the effectiveness of RT by increasing the sensitivity of cancer cells to radiation. Radiosensitizers such as PARP inhibitors and DNA repair inhibitors have been shown to enhance the abscopal effect by increasing the release of antigens and promoting an immune response [301, 302]. Targeted RT, such as radioimmunotherapy and radioligand therapy, has the potential to enhance the abscopal effect by delivering radiation to specific cancer cells [191]. Radioimmunotherapy uses monoclonal antibodies that specifically target cancer cells, while radioligand therapy uses molecules that bind to specific proteins on the surface of cancer cells. These therapies have shown promise in treating lymphoma and prostate cancer [303, 304]. However, many challenges remain for this combination treatment. The abscopal effect is a rare phenomenon, and it is difficult to predict which patients will respond to RT. Biomarkers that can predict the likelihood of an abscopal response need to be identified to optimize patient selection and treatment planning. The optimal radiation dose and timing for inducing the abscopal effect are not yet established. The effectiveness of RT can vary depending on the cancer type, stage, and location. Combining RT with immunotherapy and other therapies presents unique challenges in treatment planning and management. The timing and dosage of these therapies need to be optimized to minimize toxicities and maximize treatment effectiveness. The abscopal effect can lead to immune-related toxicities, such as autoimmune diseases and inflammation. These toxicities need to be monitored and managed to minimize their impact on patients.

The abscopal effect has the potential to revolutionize cancer treatment by providing a way to treat metastatic disease. Combining RT with immunotherapy, localized RT, radiosensitizers, and targeted RT has shown promise in enhancing the abscopal effect. However, challenges need to be addressed for successful implementation, including predicting abscopal response. It is crucial to conduct further research in the field of iRT and address the challenges discussed above before implementing it as a standardized treatment in clinical practice. More investigation is needed to fully explore the potential of iRT and overcome any barriers that may arise.

## Data Availability

No datasets were generated or analysed during the current study.
